# Tunable 3D optohydrodynamic torques from optical phase gradient–driven colloidal assemblies

**DOI:** 10.1126/sciadv.aec6957

**Published:** 2026-02-11

**Authors:** Xiao Li, Chenchen Liu, Zongpeng Huang, Jack Ng, Fan Nan

**Affiliations:** ^1^ Department of Physics and Institute for Advanced Study, The Hong Kong University of Science and Technology, Clear Water Bay, Kowloon, Hong Kong, China.; ^2^ College of Physics & Optoelectronic Engineering, Institute of Nanophotonics, Jinan University, Guangzhou 511443, China.; ^3^ Key Laboratory of Quantum Functional Materials, Department of Physics, and Guangdong Basic Research Center of Excellence for Quantum Science, Southern University of Science and Technology (SUSTech), Shenzhen 518055, Guangdong, China.

## Abstract

Optohydrodynamic manipulation offers a versatile, noninvasive, and reconfigurable approach for controlling microscopic objects. Here, we present a strategy for generating tunable three-dimensional optohydrodynamic torques through phase gradient–driven nanoparticle assemblies. Using programmable optical ring vortices (Laguerre-Gaussian beams), we assemble and rotate colloidal clusters with certain particle numbers, whose induced hydrodynamic flows apply switchable in-plane and out-of-plane torques on target particles. Torque control is achieved via two mechanisms: (i) reversing the handedness of circular polarization to break rotational symmetry and (ii) displacing optical ring vortices and modulating cluster rotation speed. These complementary controls provide robust, high-resolution torques tuned in arbitrary directions. As a proof of concept, we demonstrate full three-dimensional orientation control of a single cell. This framework greatly expands the capabilities of optohydrodynamic systems by explicitly incorporating light-driven interparticle interactions and establishes a foundation for advanced applications in biophysics, microrobotics, and biomedical engineering.

## Teaser

Optohydrodynamic control delivers 3D tunable torque via reconfigurable optical-matter chains.

## INTRODUCTION

Creating tunable and reconfigurable hydrodynamic flows using light is an emerging field of great technological importance, which enables the manipulation of particles ranging from micrometers in size down to nanometers (*1*–*15*), as well as facilitating precise molecular perturbations in intracellular studies (*16*, *17*). Such hydrodynamic flows can be generated via either static or dynamic optical driving. Static driving typically relies on optically induced mechanisms such as thermo-osmosis (*8*), thermophoresis (*18*), and Marangoni flow (*19*, *20*) under a time-independent light field. However, these approaches involve laser heating, which is generally unsuitable for temperature-sensitive biological systems. In contrast, dynamic optical driving combines optical trapping and controlled transport of microparticles (*21*–*25*), forming the basis of conventional optohydrodynamic tweezers (*3*, *4*). For instance, localized microvortices have been generated using circularly scanned optical tweezers with tightly focused laser spots (*26*, *27*). This technique has enabled the hydrodynamic rotation of cellular micromotors for targeted biocargo delivery and precision therapy (*26*).

Conventional optohydrodynamic torque (OHT) has been largely restricted to in-plane rotational control, where hydrodynamic vortices are generated with its axis perpendicular to the substrate (*3*, *10*, *26*–*28*). Such confinement arises because the optically driven microrotors or single trapped beads predominantly produce torque about the optical axis perpendicular to the field gradient. As a result, the induced motion is limited to two-dimensional circulation without the ability to reorient targets in the three-dimensional (3D) space. In contrast, out-of-plane manipulation introduces an additional degree of rotational freedom, enabling full control over particle orientation and hydrodynamic torque vectoring. This capability is particularly advantageous for 3D cell phenotyping (*29*–*35*), multiview microscopy (*36*, *37*), and reconfigurable microrobotic actuation (*38*, *39*) in confined microenvironments.

Here, we present a paradigm for OHT manipulation, introducing both conceptual and technological advances. Conceptually, we use tunable optical phase gradient–driven nanoparticle (NP) assemblies to generate substantial hydrodynamic forces on another target microparticle, with the NPs optically trapped and driven along one or two optical ring vortices (ORVs). Technologically, these ORVs, implemented by Laguerre-Gaussian beams (*40*) with programmable phase gradients and polarization handedness, enable controlled rotation of self-assembled gold NPs (Au NPs) in a two-dimensional plane near the substrate surface. This approach overcomes key limitations of traditional optohydrodynamic techniques, such as reliance on intensity gradient forces (*3*, *23*, *41*, *42*), by preventing the particle from escaping, enhancing spatial resolution, and surpassing the conventional limitation of using a single driven microparticle to create flows. Existing optical rotation techniques also involve the exploitation of Poynting vector of an optical ring, e.g., the imaginary Poynting momentum (*43*, *44*). While such mechanisms can achieve rotational motion, they typically act on individual particles and do not exploit collective hydrodynamic effects. In comparison, our phase gradient–driven colloidal assembly operates through optical momentum redistribution within a programmable ring vortex, generating switchable in-plane and out-of-plane hydrodynamic flows, which are effectively tuned by interparticle interactions. Unlike magnetic tweezers (*45*, *46*), which require functionalized magnetic particles, or acoustic tweezers (*47*), which offer lower spatial precision and necessitate more precise tuning of the acoustic field to achieve the desired trapping effect, the present approach provides label-free, reconfigurable, and field-programmable control of 3D torques using only light.

We demonstrate how variations in the number of NPs within the assembly modulate both in-plane and out-of-plane hydrodynamic rotational torques. We observe that the handedness of circular polarization plays a critical role in governing the dynamics of NP self-assembly when the sign of phase gradient is fixed. Under left-handed circularly polarized light, where the spin-converted angular momentum aligns with the phase-gradient orbital angular momentum, the particles form a continuous, uniformly spaced ring structure that collectively rotates along the optical vortex. In contrast, switching to right-handed circular polarization (RCP) induces symmetry breaking, causing the particle chain on the ORV to fragment into multiple discrete clusters, thereby disrupting its rotational symmetry. This structural asymmetry substantially influences the modulation of out-of-plane rotational torques. Furthermore, we propose an additional mechanism where NP assemblies are driven along two offset optical rings, enabling the generation of arbitrarily tunable 3D-OHTs on a microparticle. This mechanism has also been experimentally demonstrated in a more easily realizable configuration.

Our system enables both in-plane and out-of-plane robust, switchable, and tunable hydrodynamic rotations. The strength and direction of the rotational torques can be precisely adjusted by modulating the collective dynamics of the Au NP assemblies, exploiting system symmetry, or altering the strength and direction of the NPs’ velocity as they are driven by two spatially offset ORVs. These capabilities establish a versatile, noncontact, and high-precision platform for hydrodynamic manipulation, offering broad potential applications in biophysics and biomedicine.

## RESULTS

### Principle for generating in-plane and out-of-plane OHTs via orbiting colloidal assemblies

**Fig. 1.**
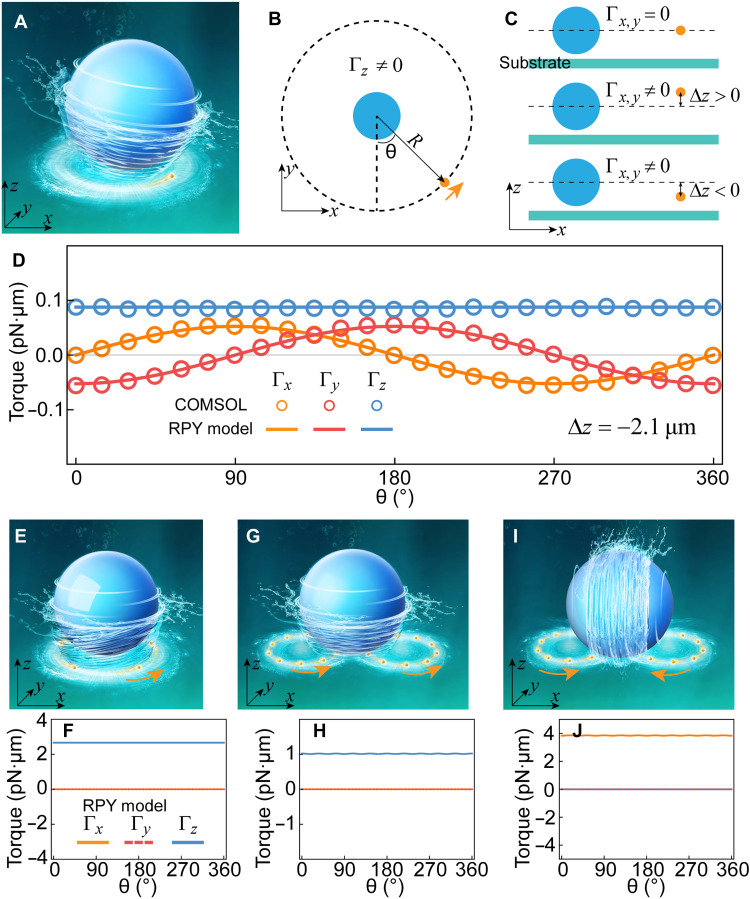
General model for creating tunable in-plane and out-of-plane optohydrodynamic rotations through in-plane optical driving of NPs. (**A** to **C**) Schematic illustrating typical hydrodynamic manipulation approaches for applying tunable hydrodynamic torque on the target particle. (A) Schematic of optohydrodynamically driven rotations. (B) The revolving Au NP generates an in-plane rotation of the blue target particle. (C) Achieving tunable and switchable out-of-plane rotations by adjusting the height of the orbiting NP. (**D**) Calculated torques applied to a target microparticle (4 μm in diameter) as a function of the azimuthal angle of the orbiting particle with a fixed ∆*z* = −2.1 μm. The diameter of the orbiting NP is 0.15 μm, with a rotation speed of 40 rounds/s and a trajectory radius *R* = 3.5 μm. Both the finite element method (COMSOL Multiphysics) and theoretical RPY model are used, and substantial agreement is demonstrated. (**E** and **F**) Six nanospheres equiangularly spaced on a circular trajectory are optically driven to revolve around the target particle (E). The generated OHTs are presented in (F). (**G** and **H**) Two groups of 10 nanospheres spaced equiangularly on two circular trajectories (each circle center offset by 3 μm) are optically driven counterclockwise (G), with the applied OHTs shown in (H). (**I** and **J**) Two groups of 10 nanospheres arranged equiangularly on two circle trajectories (with each circle center offset by 3 μm) are optically driven counterclockwise and clockwise for the left and right groups, respectively (I), with the applied torques presented in (J). The radius of the orbiting circle is *R* = 2.2 μm and ∆*z* = −2.1 μm. The orbiting speed is 40 rounds/s. Schematics in (A), (E), (G), and (I) were created using Autodesk 3ds Max.

A tunable hydrodynamic torque (Γ*_z_*) can be exerted on a target object by continuously orbiting an NP around it, as illustrated in Fig. 1A. This effect can be achieved using a scanning optical tweezers setup (*48*, *49*), where a tightly focused Gaussian beam is trapping and driving a single NP along a circular trajectory near the target particle (Fig. 1B). The resulting hydrodynamic interaction induces torque on the target particle. However, conventional strategies (*26*) typically align all particles with the same *z* position (∆*z* = 0; Fig. 1C, top panel), which substantially limits the generation of transverse torque components (Γ*_x_* and Γ*_y_*) necessary for out-of-plane rotation. This limitation is evident in movie S1, where the target particle undergoes only rotation around the *z* axis without exhibiting true 3D rotational motion.

It is found that the generation of 3D hydrodynamic torque critically depends on the axial offset ∆*z* (fig. S1). To investigate this effect, the Rotne-Prager-Yamakawa (RPY) approximation (*50*, *51*) for particles of different sizes is used to calculate the induced hydrodynamic torques

$$\left(\begin{array}{c} F_{j}\\ \Gamma_{j} \end{array}\right)=-\sum_{i}\left(\begin{array}{cc} {\overset{↔}{\zeta }}_{\mathrm{ji}}^{t-t} & {\overset{↔}{\zeta }}_{\mathrm{ji}}^{t-r}\\ {\overset{↔}{\zeta }}_{\mathrm{ji}}^{r-t} & {\overset{↔}{\zeta }}_{\mathrm{ji}}^{r-r} \end{array}\right)\left(\begin{array}{c} U_{i}\\ \Omega_{i} \end{array}\right)$$ (1)

Here, $U_{i}$ and $\Omega_{i}$ represent the translational and angular velocities of the *i*th particle, while $F_{j}=\left(F_{j,x},F_{j,y},F_{j,z}\right)$ and $\Omega_{j}=\left(\Omega_{j,x},\Omega_{j,y},\Omega_{j,z}\right)$ denote the hydrodynamic force and torque acting on the *j*th particle. The friction matrix $\left(\begin{array}{cc} {\overset{↔}{\zeta }}_{\mathrm{ji}}^{t-t} & {\overset{↔}{\zeta }}_{\mathrm{ji}}^{t-r}\\ {\overset{↔}{\zeta }}_{\mathrm{ji}}^{r-t} & {\overset{↔}{\zeta }}_{\mathrm{ji}}^{r-r} \end{array}\right)$ is defined in Materials and Methods. The translational velocity of each orbiting NP is known, while their angular velocity is considered negligible, as the spinning effect of the NPs on the opposite side cancels. Using Eq. 1, the resulting hydrodynamic forces and torques can be computed. The calculated hydrodynamic forces exerted on the target particle are small, thus with a minimal impact on its dynamics, in agreement with experimental observation.

We calculated the hydrodynamic torque acting on the target particle as a function of the angular position of the orbiting particle for ∆*z* = −2.1 μm (Fig. 1C, bottom panels) using both the RPY model and COMSOL Multiphysics simulations. The results (Fig. 1D) show that the *z* component of the torque remains constant, indicating stable in-plane spinning of the target particle. In contrast, the *x* and *y* components exhibit sinusoidal variation, confirming that the orbital motion of the smaller particle induces a genuine 3D hydrodynamic torque. It is worth noting that the COMSOL simulation includes the effect of the substrate (Fig. 1C), which is not considered in the RPY model. In addition, the two methods yield consistent results (Fig. 1D) up to a constant scaling factor applied to the RPY model.

To achieve unidirectional 3D-OHT, alternative strategies were explored. First, as shown in Fig. 1 (E and F), when multiple particles are uniformly distributed along a circular trajectory and driven synchronously around the target particle, the resulting hydrodynamic torque is directed solely along the *z* axis. Similar effects can be achieved by driving two groups of particles along separate circular trajectories, each offset by 3 μm in opposite transverse directions (Fig. 1, G and H). On the other hand, if these two groups are orbiting in opposite directions—counterclockwise on the left and clockwise on the right (Fig. 1I)—the net induced torque is oriented entirely along the transverse direction (Γ*_x_*), as shown in Fig. 1J. Γ*_z_* (Fig. 1H) and Γ*_x_* (Fig. 1J) appear to be flat, but there are still negligibly small sinusoidal fluctuations because of the fact that we have a finite number of particles (10) per trajectory. Nevertheless, the time-averaged torque clearly remains a nonzero constant, and these fluctuations can be mitigated by increasing the number of orbiting particles, as presented in fig. S2.

### Dynamics of optically driven NPs on an ORV and the induced OHT

We propose a scheme to achieve the aforementioned in-plane and out-of-plane hydrodynamic torques by continuously driving metallic NPs within ORVs. This approach enables substantial hydrodynamic torques while driving small NPs without increasing laser power.

Conventional OHT manipulation typically relies on stepwise shifting of the laser spot to induce orbital motion. However, this method produces optical intensity gradients that are insufficient to counteract fluid drag at high modulation speeds. To prevent particle escape, optical trapping forces are usually enhanced by either increasing the size of optically driven particles (at fixed power and wavelength) or raising the laser power. The former compromises spatial resolution, while the latter may induce undesired heating.

In our proposed scheme, these limitations are overcome through two key mechanisms. First, we introduce a controlled axial displacement of the NPs (Δ*z* ≠ 0), which is essential for generating reversible out-of-plane torques. Second, by modulating the optical phase gradient of the ORV, we convert axial radiation pressure into transverse driving forces, substantially enhancing the optical propulsion of NPs. This force can be finely tuned through the phase profile without altering the field’s intensity distribution (fig. S4). This capability extends beyond conventional optohydrodynamic methods, where the driving force is fundamentally constrained by the intensity-gradient optical force.

**Fig. 2.**
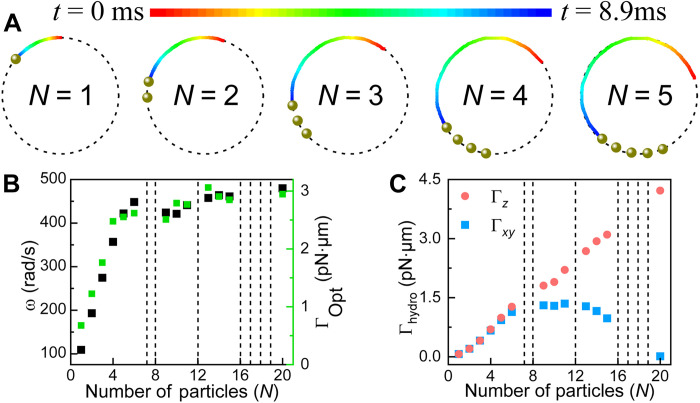
OHTs induced by ORV-driven in-plane NPs. (**A**) Configurations of *N* Au NPs, each 150 nm in diameter, driven by an ORV (Laguerre-Gaussian beam), shown for *N* = 1 to 5, starting from *t* = 0.0 ms (red) to *t* = 8.9 ms (blue). (**B**) Simulated orbital rotation speed ω (black squares) of *N* NPs in water and corresponding optical torque per NP (green squares). (**C**) Calculated hydrodynamic torque exerted on the target silica particle (Fig. 1E) as a function of *N*. Dashed lines indicate conditions under which the stable rotation of the NP cluster cannot be maintained under an ORV. Here, the ORV has an LCP and a topological charge of 1. The radius of the optical ring trap is fixed at *R* = 2.2 μm (see fig. S3 for its intensity and phase distributions).

We numerically simulated the dynamics of NPs driven by an ORV, as shown in Fig. 2A. In this configuration, multiple NPs can be trapped, self-assembled into a ridged chain structure, and propelled by phase-gradient optical forces. As the number of particles (*N*) increases, the orbital angular speed ω of the whole structure rises correspondingly (black squares in Fig. 2B) but eventually saturates. Figure 2B also shows the simulated optical torque per NP (green squares), which exhibits a similar saturation trend with *N*. Using the simulated ω, we further calculated the hydrodynamic torques exerted on the target particle (Fig. 1E) by *N* rotating NPs, presenting both the longitudinal component Γ*_z_* and the magnitude of the transverse component $\Gamma_{\mathrm{xy}}=\sqrt{\Gamma_{x}^{2}+\Gamma_{y}^{2}}$ in Fig. 2C. As expected, Γ*_z_* increases with *N*. In contrast, Γ*_xy_* first increases but then decreases to zero at *N* = 20. The finite Γ*_xy_* at small *N* arises from the nonuniform distribution of particles on the ORV, similar to the case of Fig. 1A, which breaks rotational symmetry and yields nonvanishing Γ*_x_* and Γ*_y_* (see Fig. 1D). When *N* approaches 20, the particles nearly occupy the entire ring and become equiangularly distributed, restoring approximate rotational symmetry and suppressing Γ*_x_* and Γ*_y_* to zero (Fig. 1F). Dashed lines in Fig. 2 (B and C) mark particle numbers at which the stable orbital rotation of the entire chain can no longer be sustained within the ORV, causing the chain to fragment (movie S2).

### Experimental demonstration of tunable 3D-OHTs via polarization-controlled colloidal assemblies

**Fig. 3.**
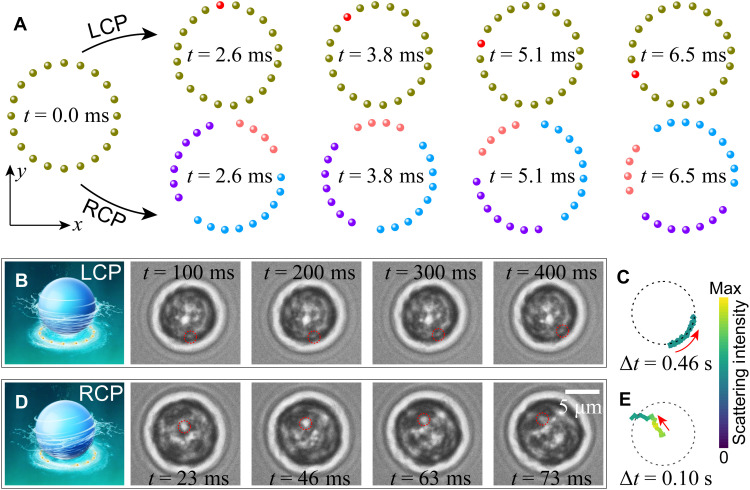
3D-OHTs induced by driving in-plane NPs with an ORV (Laguerre-Gaussian beam). (**A**) Simulated time dynamics of 20 Au NPs driven by an ORV with LCP (top row) and RCP (bottom row). Switching the polarization handedness leads to a structural rearrangement of the NP assembly. (**B** and **C**) In-plane rotation of a single yeast cell hydrodynamically driven by 20 Au NPs running on an ORV under LCP. (**D** and **E**) Out-of-plane rotation of the yeast cell hydrodynamically driven by 20 Au NPs running on an ORV under RCP. (C) and (E) show the scattering intensity traces from selected points on the cell, indicated by dashed red circles in (B) and (D), respectively. The diameter of the Au NPs is 150 nm. Schematics in (B) and (D) were created using Autodesk 3ds Max.

The dynamically reconfigurable assembly of Au NPs within a phase-gradient ORV under circular polarization is central to this work. When a circularly polarized beam is focused, its spin angular momentum is partially converted into orbital angular momentum (*52*), which drives the NPs. The conversion between spin and orbital angular momenta critically regulates the optically bound particles (*53*–*56*) and plays a key role in generating tunable hydrodynamic torques. As illustrated in the top row of Fig. 3A, under left-handed circular polarization (LCP), where the Laguerre-Gaussian beam carries a topological charge of 1, the spin-converted angular momentum aligns with the phase-gradient orbital angular momentum. Because of the lossy nature of the constituent NPs (Au), this alignment of angular momenta facilitates efficient absorption, causing the NPs to rotate in a symmetric angular configuration. It is important to emphasize that the optical torque resulting from scattering remains weak in a highly rotationally symmetric configuration (perfect rotational symmetry corresponds to zero torque) (*57*). In contrast, under RCP, the spin-converted and incident orbital angular momenta are oppositely oriented, resulting in substantially reduced absorption-induced optical torque. Nonetheless, symmetry breaking in the assembled NP structure can amplify the scattering-induced recoil torque. This amplification leads to the formation of an asymmetric configuration, ultimately causing the assembly to fragment into multiple smaller groups rather than maintaining a uniform ring structure, as depicted in the bottom row of Fig. 3A. Consequently, switching the polarization from LCP to RCP induces a structural rearrangement of the NP assembly. Moreover, the diameter of the assembled ring structure increases compared to that under LCP, indicating that the change in helicity introduces tunable radial optical force. As discussed in Fig. 2, a rotationally symmetric NP assembly produces a purely axial (Γ*_z_*) hydrodynamic torque on the target particle, whereas a rotationally asymmetric assembly generates additional transverse torque components (Γ*_x_* and Γ*_y_*). Therefore, by solely adjusting the polarization, controllable 3D-OHTs can be exerted on the target particle.

As a proof-of-concept demonstration, we used a holographic optical tweezer system (*58*, *59*). At a laser power of 10 mW, the ORV with a topological charge of 7 and a ring diameter of 4 μm drives a single Au NP to rotate at a speed up to 115 rad/s. Analysis of the beam’s *x*-*z* intensity distribution reveals that the upper part of the ORV still overlaps with the target microparticle, resulting in direct contact between the vortex field and the particle surface (fig. S5A). To suppress this effect, the ring diameter and optical power were carefully selected to prevent the direct optical rotation of the target microparticle. Furthermore, tunable OHT manipulation was demonstrated by varying the ring diameter and topological charge (fig. S5B). In all cases, the microparticle remained stably trapped at the center of the ORV and did not rotate in the absence of Au NPs (also see movie S3). Moreover, by adjusting the diameter of the ORV, we observed a clear transition from rotational to stationary behavior. This transition occurs even though the upper part of the vortex field continues to directly interact with the particle. We further demonstrate that the hydrodynamic torque exerted on the target microparticle along the *z* direction increases linearly with the number of NPs (fig. S5C). All these results provide direct experimental evidence of hydrodynamic manipulation mediated by Au NPs driven by tunable optical phase gradients.

We then focus on achieving tunable and switchable 3D-OHT control of a single yeast cell. When the number of NPs reaches 20, the ORV becomes densely populated with high rotational symmetry, causing the yeast cell to undergo pure in-plane rotation about the *z* axis (Fig. 3, B and C, and movie S4). Switching the incident polarization from left-handed to right-handed circular (LCP to RCP) (Fig. 3, D and E, and movie S5) reintroduces a hydrodynamic torque in a general 3D direction, demonstrating the tunability of our 3D-OHT manipulation strategy.

Furthermore, to achieve efficient 3D-OHTs, we also evaluate the material-dependent optical driving force on a single NP orbiting in an ORV. We identify two key parametric regions where the force is substantially enhanced, providing a practical guideline for selecting materials for phase gradient–driven hydrodynamic manipulation. High-index dielectrics (e.g., silicon and germanium) and metals (e.g., Au and Ag) emerge as optimal candidates. Conventionally, low-index particles such as silica beads are used as orbiting probes. However, our calculations show that a Au NP experiences an optical force ~10 times greater than that on a silica NP of the same size, motivating the use of Au NPs in our experiments. To optimize particle size, we compare the equivalent diameters of silica particles required to match the optical force on Au NPs (fig. S6). For small Au NPs, the ratio *d*_SiO2_/*d*_Au_ increases with NP size, but for diameters exceeding 175 nm, multipolar resonances in Au reverse this trend. Given that larger particles compromise spatial resolution, we select 150-nm Au NPs as a balance between force efficiency and resolution. The required equivalent silica size remains consistent across different phase gradients when the ORV diameter is fixed.

### Robust tunable 3D-OHTs exerted by in-plane NPs driven by two offset ORVs

**Fig. 4.**
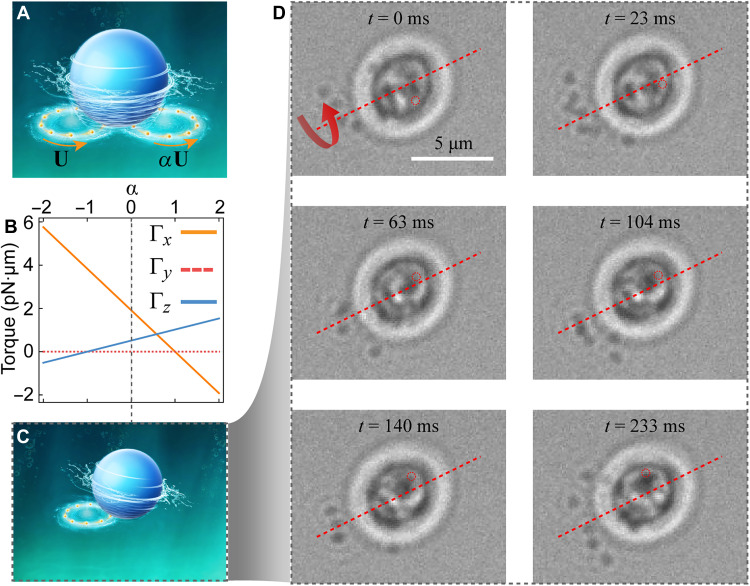
Robust tunable 3D-OHTs induced by in-plane NPs driven on two offset ORVs. (**A**) Schematic illustration of a target microparticle hydrodynamically driven by NPs rotating on two offset ORVs. The rotational velocities of NPs on the left- and right-hand rings are **U** and α**U**, respectively, where α is a tunable parameter. (**B**) Simulated hydrodynamic torques as a function of α, spanning negative to positive values. Here, the NP size, target particle size, orbiting speed of the NP, and particle positions are identical to those in Fig. 1. (**C**) Configuration for α = 0, where the NPs only orbit on the left-hand ORV, generating an out-of-plane 3D torque on the target microparticle. (**D**) Experimental realization of the configuration shown in (C). Schematics in (A) and (C) were created using Autodesk 3ds Max.

Our OHT manipulation system is highly reconfigured, allowing the 3D torque to be tuned in an arbitrary direction. As illustrated in Fig. 4A, the target microparticle could be hydrodynamically driven by in-plane NPs rotating on two ORVs offset along the *x* direction. The tangential velocities of the NPs on the left and right rings are set to **U** and α**U**, respectively, where α is a tunable parameter. The cases of α = 1 and α = −1 correspond to the configurations shown in Fig. 1 (G and I, respectively). Figure 4B presents numerical simulations of the hydrodynamic torques as α varies from −2 to 2. The results show that a pure Γ*_z_* and pure Γ*_x_* can be realized at α = 1 and α = −1, respectively. In addition, Γ*_x_* and Γ*_z_* can be tuned to positive or negative values, while Γ*_y_* remains zero. Thus, a linear combination of Γ*_x_* and Γ*_z_* enables a 3D torque along any direction within the *x*-*z* plane. Moreover, if the two ORVs are offset in both the *x* and *y* directions, a fully general 3D torque can, in principle, be generated in any spatial orientation. Experimentally, we demonstrate the 3D-OHT at α = 0, where NPs are driven on a single offset ORV, as illustrated in Fig. 4C. As shown in Fig. 4D, the yeast cell undergoes hydrodynamic rotation around the red dashed line, exhibiting clear out-of-plane spinning (also see movie S6).

Although both in-plane and out-of-plane torques coexist in the experimental configuration because of hydrodynamic coupling between neighboring flows, our simulations indicate that a pure in-plane or out-of-plane torque can be achieved in principle. Specifically, when the rotational velocities of the two offset ORVs satisfy α = 1 or α = −1, the induced hydrodynamic torque becomes unidirectional along the axial (in-plane) or transverse (out-of-plane) axis. This condition effectively decouples the axial and transverse torque components, thereby enabling pure in-plane or out-of-plane rotation, as illustrated in the configuration presented in Fig. 4B. Furthermore, the arbitrary 3D rotation is enabled by the linear superposition of hydrodynamic torques produced by multiple, independently controlled ORVs. By adjusting their relative spatial offsets, phase gradients, and angular velocities, the resultant torque vector can be continuously reoriented in the 3D space (Fig. 4B). This programmable control enables the construction of hydrodynamic torque fields of any desired orientation, extending the reach of optohydrodynamic tweezers into fully 3D manipulation.

## DISCUSSION

In summary, we presented a paradigm for hydrodynamic manipulation, leveraging optical phase gradient–driven NP assemblies to generate fully tunable 3D-OHTs. This approach overcomes the limitations of conventional optical tweezers, which rely on intensity gradient forces and single-particle orbitals. We demonstrated that dynamically reconfigurable colloidal assemblies of Au NPs within programmable ORVs can robustly generate both in-plane and out-of-plane hydrodynamic rotations. The handedness of circular polarization acts as a key control parameter, enabling precise modulation of the symmetry and dynamics of the NP assemblies. Moreover, tunable OHTs on a microparticle can, in principle, be directed in any 3D orientation by adjusting the strength and direction of phase gradient–driven NPs on two offset ORVs.

To consider potential laser-induced heating, we estimated the steady-state temperature rise around a 150-nm Au NP under an 800-nm continuous-wave laser at a light intensity of 10 mW/μm^2^ with COMSOL Multiphysics. The temperature increase near the NP is below 1 K (see fig. S7). Given the thermal diffusivity of water and the spatial separation between NPs and the target cell, the local temperature equilibrates within microseconds, ensuring negligible thermal accumulation. This modest heating confirms that the system remains compatible with temperature-sensitive biological specimens and that thermophoretic forces are insignificant compared with optical phase-gradient forces.

In addition to circular polarization, elliptically polarized light offers a continuously polarization-tunable orbital angular momentum (*60*). Such elliptical states would allow continuous tuning of the relative magnitudes of in-plane and out-of-plane hydrodynamic torques, bridging the two limiting cases of LCP and RCP. This provides a promising route for refined control of torque directionality and assembly symmetry (see fig. S8).

Through a combination of theoretical modeling, simulations, and experimental validation, we demonstrated that these NP assemblies could induce switchable and highly controllable OHTs on target objects. Our system establishes a versatile platform for contactless and high-precision manipulation at the microscale and nanoscale, paving the way for advanced applications in biophysics, biomedical manipulation, and microfluidic control.

## MATERIALS AND METHODS

### RPY approximation for particles of different sizes

Owing to the linearity of the Stokes equations, the forces ($F_{j}^{\prime }$) and torques ($\Gamma_{j}^{\prime }$) exerted on the fluid by the particles depend linearly on the translational ($U_{i}$) and rotational ($\Omega_{i}$) velocities of the particles

$$\left(\begin{array}{c} F_{j}^{\prime }\\ \Gamma_{j}^{\prime } \end{array}\right)=\sum_{i}\left(\begin{array}{cc} {\overset{↔}{\zeta }}_{\mathrm{ji}}^{t-t} & {\overset{↔}{\zeta }}_{\mathrm{ji}}^{t-r}\\ {\overset{↔}{\zeta }}_{\mathrm{ji}}^{r-t} & {\overset{↔}{\zeta }}_{\mathrm{ji}}^{r-r} \end{array}\right)\left(\begin{array}{c} U_{i}\\ \Omega_{i} \end{array}\right)$$ (2)

The reciprocal relation, which describes the particle velocities resulting from external forces and torques, is governed by the generalized mobility matrix $\left(\begin{array}{cc} {\overset{↔}{\mu }}_{\mathrm{ji}}^{t-t} & {\overset{↔}{\mu }}_{\mathrm{ji}}^{t-r}\\ {\overset{↔}{\mu }}_{\mathrm{ji}}^{r-t} & {\overset{↔}{\mu }}_{\mathrm{ji}}^{r-r} \end{array}\right)$

$$\left(\begin{array}{c} U_{i}\\ \Omega_{i} \end{array}\right)=\sum_{j}\left(\begin{array}{cc} {\overset{↔}{\mu }}_{\mathrm{ji}}^{t-t} & {\overset{↔}{\mu }}_{\mathrm{ji}}^{t-r}\\ {\overset{↔}{\mu }}_{\mathrm{ji}}^{r-t} & {\overset{↔}{\mu }}_{\mathrm{ji}}^{r-r} \end{array}\right)\left(\begin{array}{c} F_{j}^{\prime }\\ \Gamma_{j}^{\prime } \end{array}\right)$$ (3)

Here, the translational-translational mobility matrix is

$${\overset{↔}{\mu }}_{\mathrm{ji}}^{t-t}=\frac{1}{8\pi \eta R_{\mathrm{ij}}}\left[\left(1+\frac{a_{i}^{2}+a_{j}^{2}}{3R_{\mathrm{ij}}^{2}}\right)\overset{↔}{I}+\left(1-\frac{a_{i}^{2}+a_{j}^{2}}{R_{\mathrm{ij}}^{2}}\right){\hat{R}}_{\mathrm{ij}}{\hat{R}}_{\mathrm{ij}}\right]$$ (4)

the rotational-rotational mobility matrix is

$${\overset{↔}{\mu }}_{\mathrm{ji}}^{r-r}=\frac{1}{16\pi \eta R_{\mathrm{ij}}^{3}}\left[3{\hat{R}}_{\mathrm{ij}}{\hat{R}}_{\mathrm{ij}}-\overset{↔}{I}\right]$$ (5)

and the rotational-translational mobility matrix is

$${\overset{↔}{\mu }}_{\mathrm{ji}}^{r-t}=\frac{1}{8\pi \eta R_{\mathrm{ij}}^{2}}\overset{↔}{\varepsilon }\cdot {\hat{R}}_{\mathrm{ij}}$$ (6)

The translational-rotational mobility matrix (${\overset{↔}{\mu }}_{\mathrm{ji}}^{t-r}$) shares the same form with the rotational-translational one (${\overset{↔}{\mu }}_{\mathrm{ji}}^{r-t}$). In these mobility matrices, $R_{\mathrm{ij}}$ and ${\hat{R}}_{\mathrm{ij}}$ are the center-to-center distance and unit vector of $R_{\mathrm{ij}}=R_{i}-R_{j}$ for *i*th and *j*th particles, respectively, where $R_{i}$ ($R_{j}$) denotes the position of the *i*th (*j*th) particle. $a_{i}$ is the radius of the *i*th sphere, η is the viscosity of the background medium, and $\overset{↔}{I}$ and $\overset{↔}{\varepsilon }$ are the identity matrix and 3D Levi-Civita matrix, respectively.

In general, we can write Eq. 2 as

$$\left(\begin{array}{c} \tilde{U}\\ \tilde{\Omega } \end{array}\right)=\overset{↔}{\mu }\left(\begin{array}{c} {\tilde{F}}^{\prime }\\ {\tilde{\Gamma }}^{\prime } \end{array}\right)$$ (7)

with ${\tilde{F}}^{\prime }=\left(\;F_{1,x}^{\prime },F_{1,y}^{\prime },F_{1,z}^{\prime },\;\ldots ,F_{N,x}^{\prime },F_{N,y}^{\prime },F_{N,z}^{\prime }\;\right)$ being the 3 *N*-dimensional vector of external forces acting on the particles, and analogously for ${\tilde{\Gamma }}^{\prime }$, $\tilde{U}$, and $\tilde{\Omega }$. In addition, the inverse of $\overset{↔}{\mu }$ gives the friction matrix $\overset{↔}{\zeta }$. Because of the neglect of inertia, the external forces ${\tilde{F}}^{\prime }$ and torques ${\tilde{\Gamma }}^{\prime }$ acting on the particles are exactly balanced by the hydrodynamic forces and torques exerted by the fluid. Consequently, the hydrodynamic forces and torques on the particles are equal in magnitude and opposite in direction to the applied external forces and torques

$$\left(\begin{array}{c} \tilde{F}\\ \tilde{\Gamma } \end{array}\right)=-\left(\begin{array}{c} {\tilde{F}}^{\prime }\\ {\tilde{\Gamma }}^{\prime } \end{array}\right)$$ (8)

One can obtain Eq. 1 with Eqs. 2 and 8.

### Numerical simulation

The fluid field distribution was simulated using the finite element method with the Rotating Machinery, Laminar Flow module in COMSOL Multiphysics. A no-slip boundary condition was applied at all chamber surfaces. To reduce the influence of lateral boundaries on the internal microvortex while keeping the computational load manageable, the chamber length and width were set to 500 μm. The chamber height was set to 120 μm to match the experimental setup, ensuring that the simulation closely reflected actual conditions. The target particle and orbiting particle were both positioned near the bottom surface of the chamber, consistent with their locations during experiments. The hydrodynamic torques acting on the particles were calculated by integrating the shear stress over the particle surfaces using COMSOL’s built-in postprocessing tools.

The hologram required to generate the ORV trap was calculated using a previously developed method, enabling precise reconstruction of the desired optical field. The transverse electric field components, E*_x_* and E*_y_*, were obtained and imported into the finite-difference time-domain (FDTD) software via the Import Source function. NPs were positioned in the *x*-*y* plane at fixed *z* coordinates and immersed in water. Optical forces acting on each particle were calculated using the Maxwell stress tensor. To investigate the dynamic behavior of the NPs within the ORV, a coupled FDTD-Langevin dynamics approach was used. In this method, optical forces calculated from the FDTD simulations were used to determine NP velocities, which in turn updated particle positions according to the two-dimensional Langevin equations. This iterative process allowed for the prediction of NP trajectories over time, with optical forces recorded at each time step for detailed analysis.

### Experiment setup

The holographic optical tweezers system used in our experiments consists of a continuous-wave laser (Spectra-Physics 3900S, operated at 800 nm), a spatial light modulator (Hamamatsu X15213-02), and a custom-built microscope. The sample cell was assembled by sandwiching a 100-μm-thick spacer between two coverslips (ISOLAB), creating a sealed chamber filled with an aqueous suspension of nano- or microparticles. Particle dynamics were visualized using bright-field microscopy with a water-immersion objective (Olympus UPLSAPO 60XW) and recorded at 200 frames per second with a complementary metal-oxide semiconductor camera (Point Grey Grasshopper3).

### Materials

Colloidal solutions of Au NPs were purchased from Sigma-Aldrich and further diluted with deionized water. Silica microparticles (5 μm in diameter) were purchased from Huge Biotech Co., Ltd. (Shanghai, China). Yeast cells (ATCC 9763) were purchased from Shanghai Luwei Technology Co., Ltd., and cultured in YPD medium (yeast extract, peptone, and dextrose) at pH 4.5 and 26°C. After cultivation, the yeast cells were washed and resuspended in phosphate-buffered saline to achieve a concentration of ~3.0 × 10^3^ cells μl^−1^.

### Scalability

To further clarify the scalability of this method, the central particle is ideally microsized, with diameters ranging from 1 μm to several tens of micrometers. For smaller particles, the optical ring size can be adjusted to maintain effective rotational control. However, in the nanoscale regime, the size of the optical ring cannot be further reduced because of its angular momentum and diffraction limit, and the central particle tends to be attracted to the optical ring region, which may limit the method’s applicability in achieving controlled rotation at such scales.

## References

[1] D. Psaltis, S. R. Quake, C. Yang, Developing optofluidic technology through the fusion of microfluidics and optics. Nature 442, 381–386 (2006). 16871205 10.1038/nature05060

[2] L. Lin, M. Wang, X. Peng, E. N. Lissek, Z. Mao, L. Scarabelli, E. Adkins, S. Coskun, H. E. Unalan, B. A. Korgel, L. M. Liz-Marzán, E.-L. Florin, Y. Zheng, Opto-thermoelectric nanotweezers. Nat. Photonics 12, 195–201 (2018). 29785202 10.1038/s41566-018-0134-3 PMC5958900

[3] U. G. Būtaitė, G. M. Gibson, Y.-L. D. Ho, M. Taverne, J. M. Taylor, D. B. Phillips, Indirect optical trapping using light driven micro-rotors for reconfigurable hydrodynamic manipulation. Nat. Commun. 10, 1215 (2019). 30872572 10.1038/s41467-019-08968-7 PMC6418258

[4] J. Chen, J. F.-C. Loo, D. Wang, Y. Zhang, S.-K. Kong, H.-P. Ho, Thermal optofluidics: Principles and applications. Adv. Opt. Mater. 8, 1900829 (2020).

[5] Z. Chen, J. Li, Y. Zheng, Heat-mediated optical manipulation. Chem. Rev. 122, 3122–3179 (2021). 34797041 10.1021/acs.chemrev.1c00626 PMC9833329

[6] F. Schmidt, H. Šípová-Jungová, M. Käll, A. Würger, G. Volpe, Non-equilibrium properties of an active nanoparticle in a harmonic potential. Nat. Commun. 12, 1902 (2021). 33772007 10.1038/s41467-021-22187-z PMC7998004

[7] B. Ciraulo, J. Garcia-Guirado, I. de Miguel, J. Ortega Arroyo, R. Quidant, Long-range optofluidic control with plasmon heating. Nat. Commun. 12, 2001 (2021). 33790293 10.1038/s41467-021-22280-3 PMC8012589

[8] M. Fränzl, F. Cichos, Hydrodynamic manipulation of nano-objects by optically induced thermo-osmotic flows. Nat. Commun. 13, 656 (2022). 35115502 10.1038/s41467-022-28212-z PMC8813924

[9] S. Yang, J. C. Ndukaife, Optofluidic transport and assembly of nanoparticles using an all-dielectric quasi-BIC metasurface. Light Sci. Appl. 12, 188 (2023). 37507389 10.1038/s41377-023-01212-4 PMC10382587

[10] X. Wang, P.-C. Chen, K. Kroy, V. Holubec, F. Cichos, Spontaneous vortex formation by microswimmers with retarded attractions. Nat. Commun. 14, 56 (2023). 36599830 10.1038/s41467-022-35427-7 PMC9813373

[11] J. Xiong, Y. Shi, T. Pan, D. Lu, Z. He, D. Wang, X. Li, G. Zhu, B. Li, H. Xin, Wake-riding effect-inspired opto-hydrodynamic diatombot for non-invasive trapping and removal of nano-biothreats. Adv. Sci. 10, 2301365 (2023). 10.1002/advs.202301365 PMC10288256 37012610

[12] F. Schmidt, C. D. González-Gómez, M. Sulliger, E. Ruiz-Reina, R. A. Rica-Alarcón, J. Ortega Arroyo, R. Quidant, Three-dimensional optofluidic control using reconfigurable thermal barriers. Nat. Photonics 19, 1385–1391 (2025). 41346951 10.1038/s41566-025-01731-z PMC12672375

[13] M. Yang, Y. Shi, Q. Song, Z. Wei, X. Dun, Z. Wang, Z. Wang, C.-W. Qiu, H. Zhang, X. Cheng, Optical sorting: Past, present and future. Light Sci. Appl. 14, 103 (2025). 40011460 10.1038/s41377-024-01734-5 PMC11865320

[14] Y. Peng, J. Zhou, J. Chen, P. Du, Z. Jin, X. Dai, Y. Zhong, Y. Ji, Z. Chen, M. Wang, Y. Wang, A. H.-P. Ho, S. Zeng, Q. Jiang, L. Ma, O. G. Schmidt, Y. Zheng, J. Qu, Y. Shao, Optothermal ice–water interface management for cross-scale enrichment and molecular sensing. ACS Nano 19, 39281–39291 (2025). 41201957 10.1021/acsnano.5c13123

[15] Y. Yang, K. Jin, H. Qi, W. Wei, Y. Zhou, C. Sun, X. Chen, Y. Wang, L. P. Lee, X. Duan, Acoustic streaming microgripper for programmable three-dimensional manipulation of single cells. NPJ Acoust. 1, 19 (2025).

[16] M. Mittasch, P. Gross, M. Nestler, A. W. Fritsch, C. Iserman, M. Kar, M. Munder, A. Voigt, S. Alberti, S. W. Grill, M. Kreysing, Non-invasive perturbations of intracellular flow reveal physical principles of cell organization. Nat. Cell Biol. 20, 344–351 (2018). 29403036 10.1038/s41556-017-0032-9

[17] H.-Y. Wu, G. Kabacaoğlu, E. Nazockdast, H.-C. Chang, M. J. Shelley, D. J. Needleman, Laser ablation and fluid flows reveal the mechanism behind spindle and centrosome positioning. Nat. Phys. 20, 157–168 (2024).

[18] S. Liu, L. Lin, H.-B. Sun, Opto-thermophoretic manipulation. ACS Nano 15, 5925–5943 (2021). 33734695 10.1021/acsnano.0c10427

[19] C. Lv, S. N. Varanakkottu, T. Baier, S. Hardt, Controlling the trajectories of nano/micro particles using light-actuated marangoni flow. Nano Lett. 18, 6924–6930 (2018). 30285458 10.1021/acs.nanolett.8b02814

[20] P. Dara, M. Käll, Bubble dynamics and directional marangoni flow induced by laser heating of silicon nanodisk arrays. J. Phys. Chem. C 129, 5502–5510 (2025).

[21] A. Ashkin, J. M. Dziedzic, J. E. Bjorkholm, S. Chu, Observation of a single-beam gradient force optical trap for dielectric particles. Opt. Lett. 11, 288–290 (1986). 19730608 10.1364/ol.11.000288

[22] D. Gao, W. Ding, M. Nieto-Vesperinas, X. Ding, M. Rahman, T. Zhang, C. Lim, C.-W. Qiu, Optical manipulation from the microscale to the nanoscale: Fundamentals, advances and prospects. Light Sci. Appl. 6, e17039 (2017). 30167291 10.1038/lsa.2017.39 PMC6062326

[23] F. Nan, Z. Yan, Optical sorting at the single-particle level with single-nanometer precision using coordinated intensity and phase gradient forces. ACS Nano 14, 7602–7609 (2020). 32428394 10.1021/acsnano.0c03478

[24] L.-M. Zhou, Y. Shi, X. Zhu, G. Hu, G. Cao, J. Hu, C.-W. Qiu, Recent progress on optical micro/nanomanipulations: Structured forces, structured particles, and synergetic applications. ACS Nano 16, 13264–13278 (2022). 36053722 10.1021/acsnano.2c05634

[25] X. Li, Y. Yang, S. Yan, W. Gao, Y. Zhou, X. Yu, C. Bai, D. Dan, X. Xu, B. Yao, Artificial potential field-empowered dynamic holographic optical tweezers for particle-array assembly and transformation. PhotoniX 5, 32 (2024).

[26] X. Zou, Q. Zheng, D. Wu, H. Lei, Controllable cellular micromotors based on optical tweezers. Adv. Funct. Mater. 30, 2002081 (2020).

[27] Y. Zhao, X. Liu, Z. Gong, J. Xu, T. Wu, H. Wu, J. Guo, Y. Li, B. Li, Y. Zhang, Light-driven micronavigators for directional migration of cells. Laser Photonics Rev. 18, 2400058 (2024).

[28] I. Williams, E. C. Oğuz, T. Speck, P. Bartlett, H. Löwen, C. P. Royall, Transmission of torque at the nanoscale. Nat. Phys. 12, 98–103 (2016).

[29] D. Ahmed, A. Ozcelik, N. Bojanala, N. Nama, A. Upadhyay, Y. Chen, W. Hanna-Rose, T. J. Huang, Rotational manipulation of single cells and organisms using acoustic waves. Nat. Commun. 7, 11085 (2016). 27004764 10.1038/ncomms11085 PMC4814581

[30] Y. Liu, H. Ding, J. Li, X. Lou, M. Yang, Y. Zheng, Light-driven single-cell rotational adhesion frequency assay. eLight 2, 13 (2022). 35965781 10.1186/s43593-022-00020-4 PMC9358104

[31] L. Shen, Z. Tian, K. Yang, J. Rich, J. Xia, N. Upreti, J. Zhang, C. Chen, N. Hao, Z. Pei, T. J. Huang, Joint subarray acoustic tweezers enable controllable cell translation, rotation, and deformation. Nat. Commun. 15, 9059 (2024). 39428395 10.1038/s41467-024-52686-8 PMC11491459

[32] X. Xu, M. Nieto-Vesperinas, Y. Zhou, Y. Zhang, M. Li, F. J. Rodríguez-Fortuño, S. Yan, B. Yao, Gradient and curl optical torques. Nat. Commun. 15, 6230 (2024). 39043631 10.1038/s41467-024-50440-8 PMC11266349

[33] X. Cui, V. Mylnikov, P. Johansson, M. Käll, Synchronization of optically self-assembled nanorotors. Sci. Adv. 10, eadn3485 (2024). 38457509 10.1126/sciadv.adn3485 PMC10923511

[34] Y.-J. Wu, J.-H. Zhuang, P.-P. Yu, Y.-F. Liu, Z.-Q. Wang, Y.-M. Li, C.-W. Qiu, L. Gong, Time-varying 3D optical torque via a single beam. Nat. Commun. 16, 593 (2025). 39799144 10.1038/s41467-024-55781-y PMC11724974

[35] G. Wang, M. Rey, A. Ciarlo, M. Shanei, K. Xiong, G. Pesce, M. Käll, G. Volpe, Microscopic geared mechanisms. Nat. Commun. 16, 7767 (2025). 40835594 10.1038/s41467-025-62869-6 PMC12368165

[36] B. Yang, M. Lange, A. Millett-Sikking, X. Zhao, J. Bragantini, S. VijayKumar, M. Kamb, R. Gómez-Sjöberg, A. C. Solak, W. Wang, H. Kobayashi, M. N. McCarroll, L. W. Whitehead, R. P. Fiolka, T. B. Kornberg, A. G. York, L. A. Royer, DaXi—High-resolution, large imaging volume and multi-view single-objective light-sheet microscopy. Nat. Methods 19, 461–469 (2022). 35314838 10.1038/s41592-022-01417-2 PMC9007742

[37] O. Zhang, Z. Guo, Y. He, T. Wu, M. D. Vahey, M. D. Lew, Six-dimensional single-molecule imaging with isotropic resolution using a multi-view reflector microscope. Nat. Photonics 17, 179–186 (2023). 36968242 10.1038/s41566-022-01116-6 PMC10035538

[38] X. Wu, R. Ehehalt, G. Razinskas, T. Feichtner, J. Qin, B. Hecht, Light-driven microdrones. Nat. Nanotechnol. 17, 477–484 (2022). 35449413 10.1038/s41565-022-01099-z

[39] X. Li, J. Ng, Microdrones soar by recoiling light. Nat. Nanotechnol. 17, 438–439 (2022). 35449412 10.1038/s41565-022-01094-4

[40] A. T. O’Neil, I. MacVicar, L. Allen, M. J. Padgett, Intrinsic and extrinsic nature of the orbital angular momentum of a light beam. Phys. Rev. Lett. 88, 053601 (2002). 11863722 10.1103/PhysRevLett.88.053601

[41] X. Xu, M. Nieto-Vesperinas, C.-W. Qiu, X. Liu, D. Gao, Y. Zhang, B. Li, Kerker-type intensity-gradient force of light. Laser Photonics Rev. 14, 1900265 (2020).

[42] H. Zheng, H. Chen, J. Ng, Z. Lin, Optical gradient force in the absence of light intensity gradient. Phys. Rev. B 103, 035103 (2021).

[43] Y. Zhou, X. Xu, Y. Zhang, M. Li, S. Yan, M. Nieto-Vesperinas, B. Li, C.-W. Qiu, B. Yao, Observation of high-order imaginary Poynting momentum optomechanics in structured light. Proc. Natl. Acad. Sci. U.S.A. 119, e2209721119 (2022). 36279457 10.1073/pnas.2209721119 PMC9636969

[44] F. Nan, X. Li, S. Huang, S. Zhang, J. Ng, Y. Zheng, Tunable photon-recoil forces and negative torque at flat-top beam edges. Nat. Commun. 16, 9342 (2025). 41125587 10.1038/s41467-025-64423-w PMC12546578

[45] K. C. Neuman, A. Nagy, Single-molecule force spectroscopy: Optical tweezers, magnetic tweezers and atomic force microscopy. Nat. Methods 5, 491–505 (2008). 18511917 10.1038/nmeth.1218 PMC3397402

[46] I. D. Vlaminck, C. Dekker, Recent advances in magnetic tweezers. Annu. Rev. Biophys. 41, 453–472 (2012). 22443989 10.1146/annurev-biophys-122311-100544

[47] A. Ozcelik, J. Rufo, F. Guo, Y. Gu, P. Li, J. Lata, T. J. Huang, Acoustic tweezers for the life sciences. Nat. Methods 15, 1021–1028 (2018). 30478321 10.1038/s41592-018-0222-9 PMC6314293

[48] K. Sasaki, M. Koshioka, H. Misawa, N. Kitamura, H. Masuhara, Optical trapping of a metal particle and a water droplet by a scanning laser beam. Appl. Phys. Lett. 60, 807–809 (1992).

[49] L. A. Shaw, R. M. Panas, C. M. Spadaccini, J. B. Hopkins, Scanning holographic optical tweezers. Opt. Lett. 42, 2862–2865 (2017). 28957193 10.1364/OL.42.002862

[50] E. Wajnryb, K. A. Mizerski, P. J. Zuk, P. Szymczak, Generalization of the Rotne–Prager–Yamakawa mobility and shear disturbance tensors. J. Fluid Mech. 731, R3 (2013).

[51] P. J. Zuk, E. Wajnryb, K. A. Mizerski, P. Szymczak, Rotne–Prager–Yamakawa approximation for different-sized particles in application to macromolecular bead models. J. Fluid Mech. 741, R5 (2014).

[52] T. A. Nieminen, A. B. Stilgoe, N. R. Heckenberg, H. Rubinsztein-Dunlop, Angular momentum of a strongly focused Gaussian beam. J. Opt. A Pure Appl. Opt. 10, 115005 (2008).

[53] M. Tamura, T. Omatsu, S. Tokonami, T. Iida, Interparticle-interaction-mediated anomalous acceleration of nanoparticles under light-field with coupled orbital and spin angular momentum. Nano Lett. 19, 4873–4878 (2019). 31272154 10.1021/acs.nanolett.9b00332

[54] J. Parker, S. Nagasamudram, C. Peterson, Y. Li, S. Soleimanikahnoj, S. A. Rice, N. F. Scherer, Symmetry breaking-induced N-body electrodynamic forces in optical matter systems. Nat. Commun. 16, 6294 (2025). 40628715 10.1038/s41467-025-61616-1 PMC12238463

[55] F. Nan, X. Li, S. Zhang, J. Ng, Z. Yan, Creating stable trapping force and switchable optical torque with tunable phase of light. Sci. Adv. 8, eadd6664 (2022). 36399578 10.1126/sciadv.add6664 PMC9674277

[56] F. Nan, F. Han, N. F. Scherer, Z. Yan, Dissipative self-assembly of anisotropic nanoparticle chains with combined electrodynamic and electrostatic interactions. Adv. Mater. 30, 1803238 (2018). 10.1002/adma.201803238 30239041

[57] J. Chen, J. Ng, K. Ding, K. H. Fung, Z. Lin, C. T. Chan, Negative optical torque. Sci. Rep. 4, 6386 (2014). 25226863 10.1038/srep06386 PMC4165981

[58] J. E. Curtis, B. A. Koss, D. G. Grier, Dynamic holographic optical tweezers. Opt. Commun. 207, 169–175 (2002).

[59] J. Leach, G. Sinclair, P. Jordan, J. Courtial, M. J. Padgett, J. Cooper, Z. J. Laczik, 3D manipulation of particles into crystal structures using holographic optical tweezers. Opt. Express 12, 220–226 (2004). 19471528 10.1364/opex.12.000220

[60] Y. Shi, T. Zhu, A. Q. Liu, L.-M. Zhou, M. Nieto-Vesperinas, A. Hassanfiroozi, J. Liu, D. P. Tsai, Z. Li, W. Ding, F. Wang, H. Li, Q. Song, X. Xu, B. Li, X. Cheng, P. C. Wu, C. T. Chan, C.-W. Qiu, Inverse optical torques on dielectric nanoparticles in elliptically polarized light waves. Phys. Rev. Lett. 129, 053902 (2022). 35960581 10.1103/PhysRevLett.129.053902

