# Supplementary material for: Tunable 3D optohydrodynamic torques from optical phase gradient–driven colloidal assemblies
Source: Sci Adv. 2026 Feb 11;12(7):eaec6957. doi: 10.1126/sciadv.aec6957 (PMC12893283; doi:10.1126/sciadv.aec6957)
Supplement: Supplementary file 1 — Figs. S1 to S8 [file sciadv.aec6957_sm.pdf]

Science Advances
AAAS

Supplementary Materials for

Tunable 3D optohydrodynamic torques from optical phase gradient–driven colloidal assemblies

Xiao Li *et al.*

Corresponding author: Jack Ng, wuzh3@sustech.edu.cn; Fan Nan, fnan190730@gmail.com

Sci. Adv. **12**, eaec6957 (2026)
DOI: 10.1126/sciadv.aec6957

The PDF file includes:

Figs. S1 to S8

Other Supplementary Material for this manuscript includes the following:

Movies S1 to S6

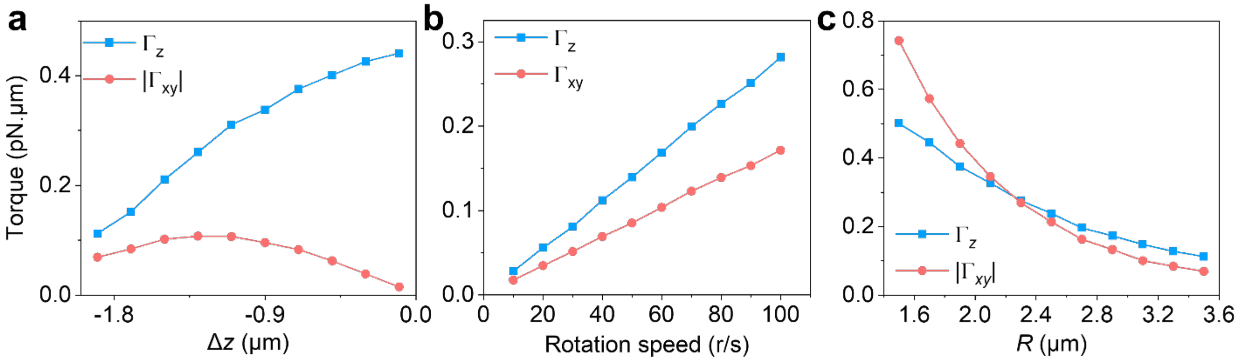

Supplementary Figure S1. Principle of creating in-plane and out-of-plane hydrodynamic rotational torques. (a) Calculated torques applied on a target microparticle (4 μm in dia.) as a function of the center-to-center distances (Δ*z*) of the two particles along *z*-axis. Γ_z and Γ_{xy} denote the torques that generate in-plane and out-of-plane rotational motions, respectively. $\Gamma_{xy} = \sqrt{\Gamma_x^{\ 2} + \Gamma_y^{\ 2}}$, representing the magnitude of the in-plane hydrodynamic torque. The diameter of the orbiting NP is fixed at 200 nm. The rotation speed and radius of the NP is 40 r/s and 3.5 μm, respectively. Tuning in-plane and out-of-plane torques by modulating the rotation speed (b) and radius (c) of the orbiting NP. In these cases, Δ*z* is fixed at 1.9 μm.

The mechanism of creating in-plane and out-of-plane hydrodynamic rotational torques is analogous to the spin generation in table tennis: top-spin or back-spin is imparted depending on whether the paddle strikes the upper or lower part of the ball. We calculated the hydrodynamic torques (Γ_z and Γ_{xy}) exerted on a single SiO_2 microparticle as a function of the axial offset Δ*z* (Supplementary Figure S1a). The torque (Γ_z) responsible for in-plane rotation is generally stronger than the torque (Γ_{xy}) that induces out-of-plane rotation across different Δ*z* values. When $\Delta z \neq 0$, both torque components can be enhanced by increasing the orbital speed of the nanoparticle (Supplementary Figure S1b). Furthermore, the orbital radius (R) serves as a tunable parameter that, under a fixed Δ*z*, enables selective enhancement of either the in-plane or out-of-plane rotation (Supplementary Figure S1c). Specifically, by adjusting *R*, Γ_{xy} can be made to exceed Γ_z, providing additional control over the rotational behavior of the system.

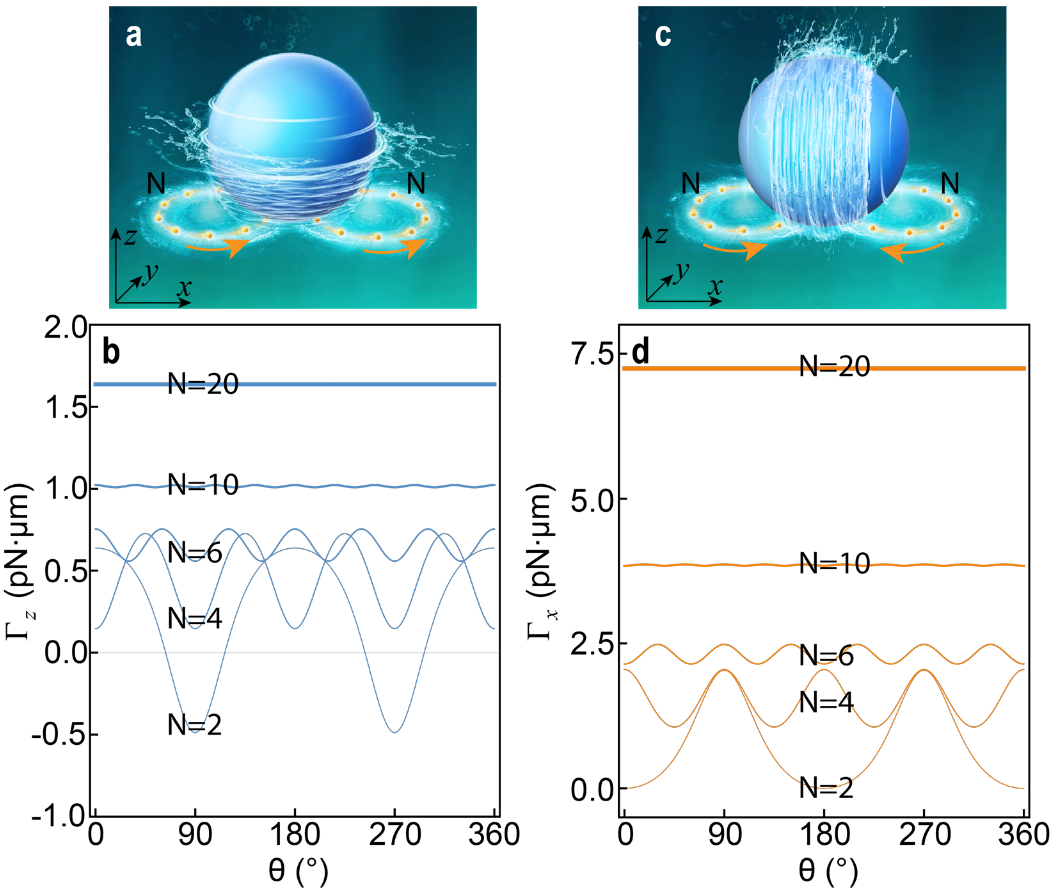

Supplementary Figure S2. Hydrodynamic torques for different numbers of orbiting nanoparticles (N) driven on each optical ring vortex. (a) Nanoparticles driven in the same direction. (b) Corresponding hydrodynamic torques as the nanoparticle orientation (θ) varies. (c) Nanoparticles driven in opposite directions. (d) Corresponding hydrodynamic torques as the nanoparticle orientation (θ) varies. The induced hydrodynamic torques gradually approach invariance as N increases. Schematics in (a) and (c) were created using Autodesk 3ds Max.

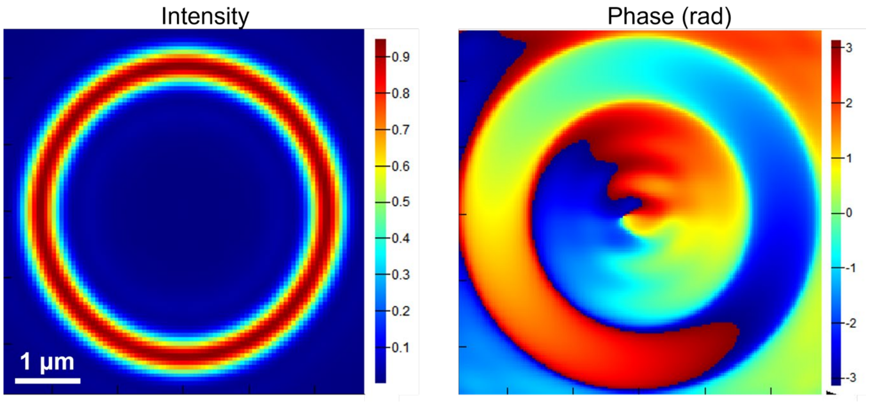

Supplementary Figure S3. Intensity and phase profiles of a left-hand circularly polarized Laguerre–Gaussian beam with a 4.4 μm diameter and a topological charge of 1.

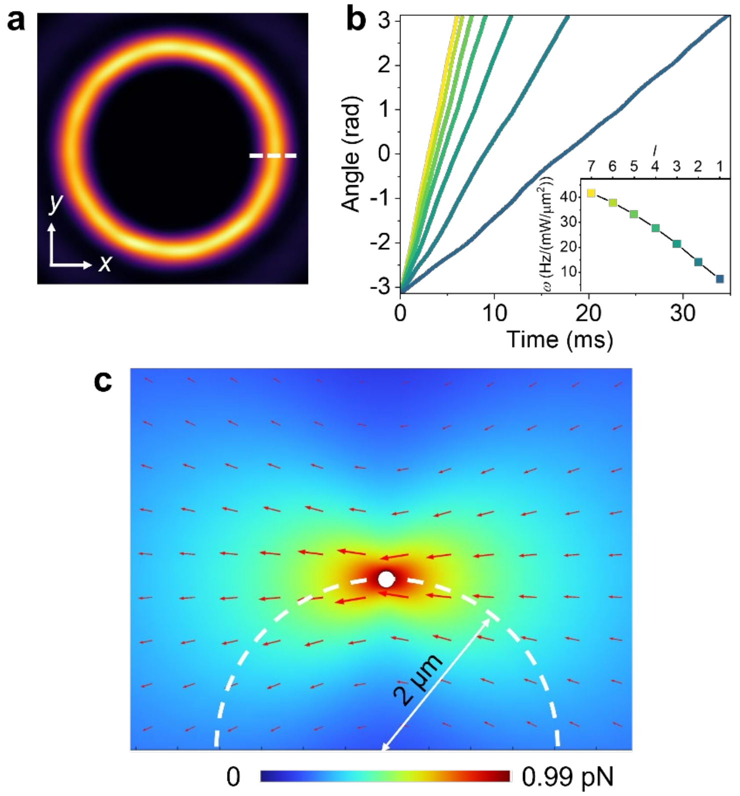

Supplementary Figure S4. Opto-hydrodynamic manipualtion based on phase-gradient-driven Au nanoparticles. (a) Simulated intensity profile of the ring trap. (b) Simulated angular trajectories of a single Au NP driven by a series of ring trap with different topological charges (*l*). The laser intensity is fixed at 4 mW/μm^2. The inset shows the calculated angular velocity. Dynamic FDTD simulations confirm that the nanoparticle rotation speed increases with topological charge at constant laser intensity. At *l* = 7, the system reaches a rotation frequency of 40 Hz/(mW/μm^2), generating substantial hydrodynamic shear forces. (c) The simulated distribution of the hydrodynamic forces created by a single Au NP in its rotational plane. The diameter of the ring is 4 μm and the rotation speed of the NP is 45 rounds per second.

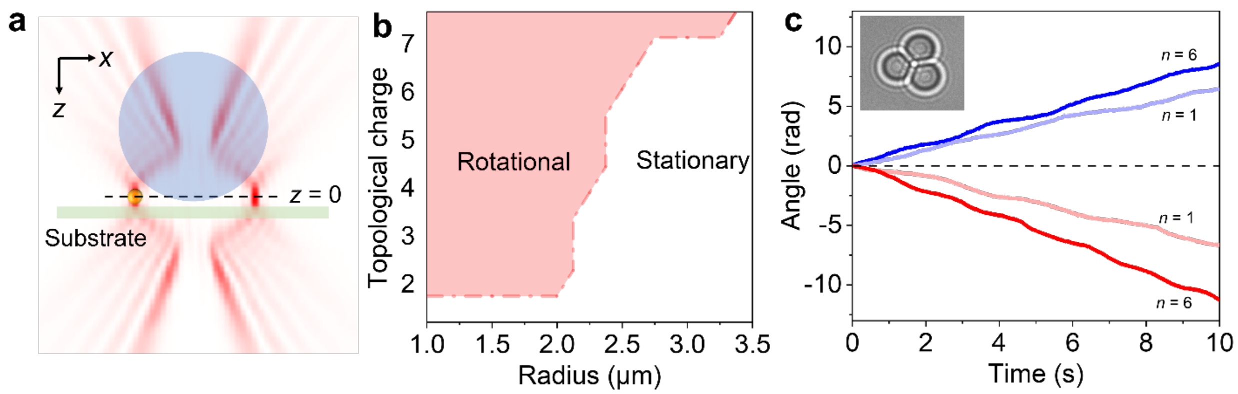

Supplementary Figure S5. Experimental demonstration of opto-hydrodynamic manipulation via phase-gradient-driven metal nanoparticles. (a) Illustration of the experimental setup and *x*-*z* view of the optical ring. The dashed line represents the manipulation plane of Au NPs (*x*-*y* plane at *z* = 0). The optical field propagated along the +*z* direction. The diameters of the optical ring and silica microparticle are 4 and 5 μm, respectively. (b) Phase diagram for rotating and stopping of a single silica microparticle by tuning of the diameter and topological charge of the optical ring. (c) Measured angular trajectories of a self-assembled silica cluster. With a fixed laser power, direction-switchable and magnitude-tunable hydrodynamic rotation is readily achieved by modulating the sign of topological charge and number (*n*) of optical-driven Au NPs. The topological charge is switched between 3 and -3.

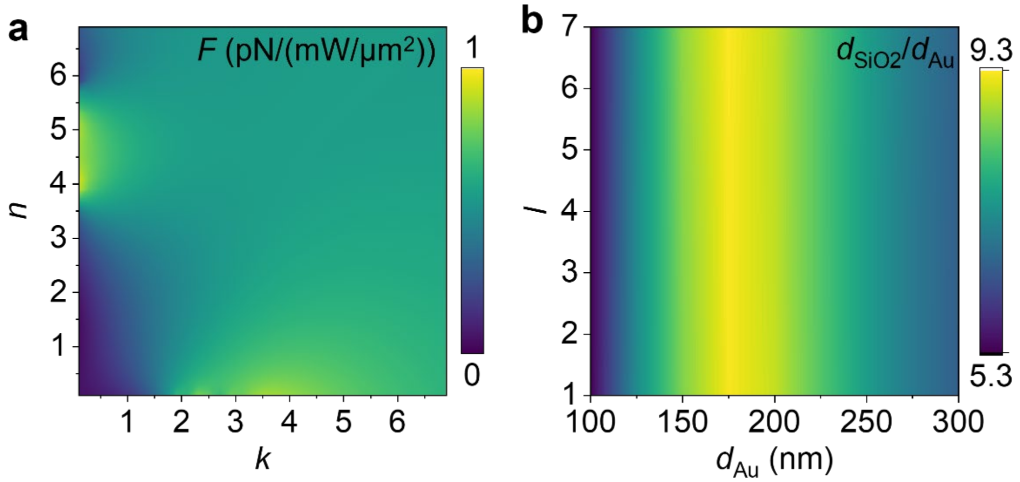

Supplementary Figure S6. Guidelines for selecting materials and particle sizes in colloidal assemblies for phase-gradient-driven hydrodynamic manipulation. (a) Calculated optical phase gradient force applied on a single NP with different materials. The diameter of the optical ring and NP are fixed at 4.4 and 0.2 μm, respectively. The laser wavelength is 800 nm. (b) The required diameters of silica NP (d_{SiO2}) in generating optical force with the same magnitude with respect to Au NP of different sizes (d_{Au}) at different topological charge (*l*).

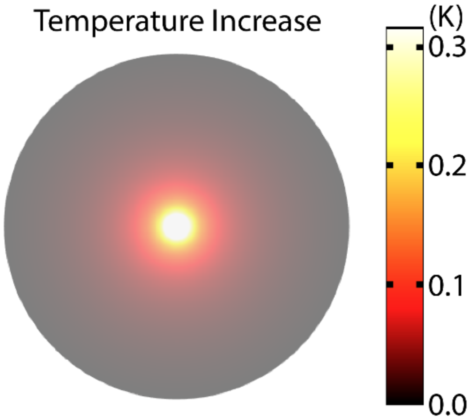

Supplementary Figure S7. Temperature increase of the gold sphere with a diameter of 150 nm when illuminated by a plane wave with an intensity of 10 mW/μm² and a wavelength of 800 nm.

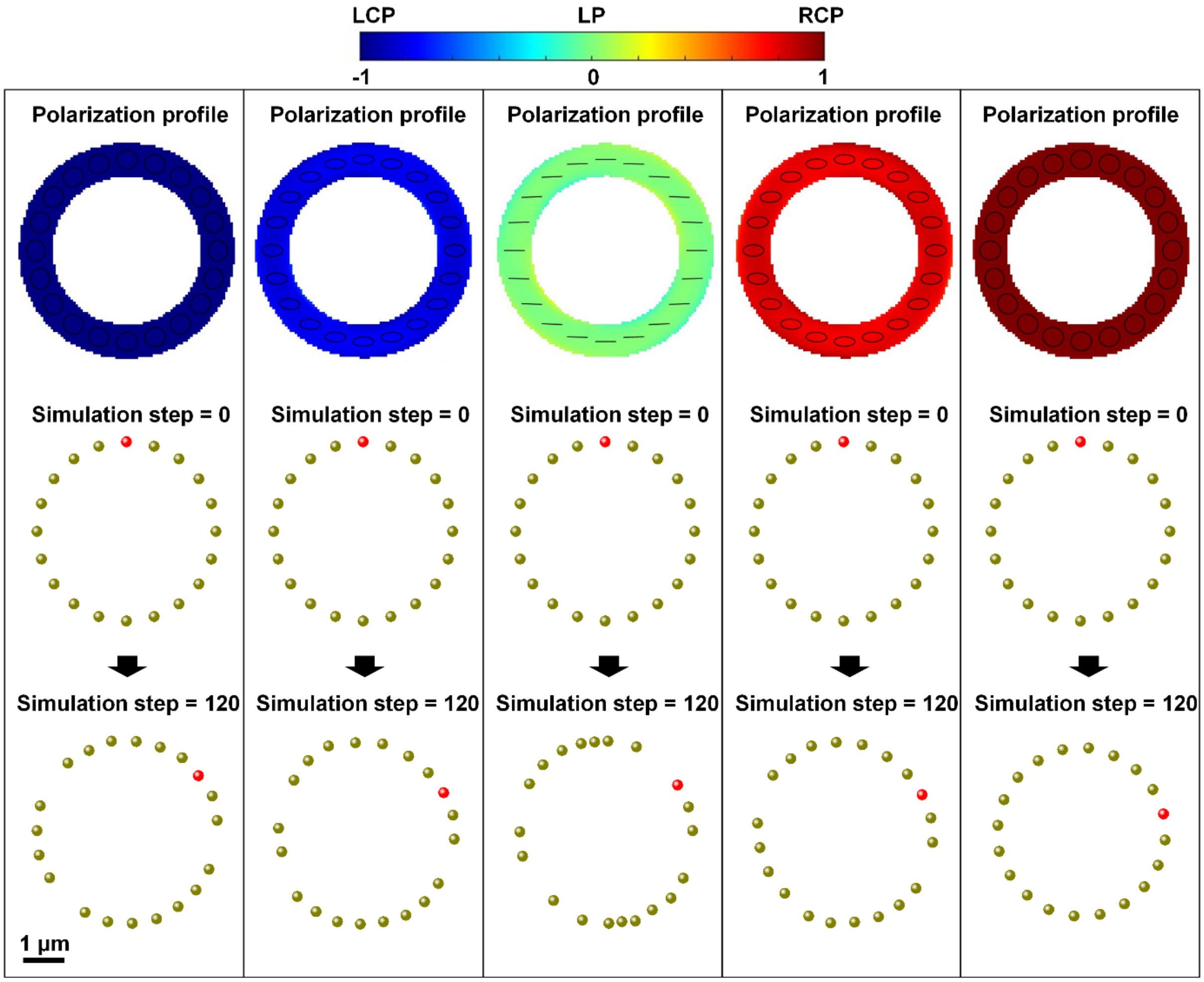

Supplementary Figure S8. Influence of elliptically polarized light on nanoparticle assembly. The optical field carries a topological charge of -1, and the phase-gradient direction is clockwise. In all

simulations, 20 identical gold nanoparticles (diameter 150 nm) are used. After 120 simulation steps, distinct assembly configurations emerge depending on the polarization state. We find that only under left- or right-circular polarization (LCP or RCP) do the particles reach a stable configuration within 120 steps. In these cases, the phase-gradient direction is opposite to that shown in the main figure, leading to uniform inter-particle separation under RCP illumination. Furthermore, under elliptical polarization the particles assemble into an elliptical ring, and this geometry enables additional tunability in the resulting hydrodynamic torque.
